# Effects of Integrated Fuzzy Logic PID Controller on Satellite Antenna Tracking System

**DOI:** 10.1155/2022/7417298

**Published:** 2022-03-07

**Authors:** R. Obulakonda Reddy, Sandeep Kautish, V. Padmanabha Reddy, N. Sudhakar Yadav, Meznah M. Alanazi, Ali Wagdy Mohamed

**Affiliations:** ^1^Department of Computer Science and Engineering, Institute of Aeronautical Engineering, Dundigal, Hyderabad-97, India; ^2^LBEF Campus, Kathmandu 44600, Nepal; ^3^Department of ECE, Institute of Aeronautical Engineering, Dundigal, Hyderabad-97, India; ^4^Deptartment of Information Technology, VNR Vignana Jyothi Institute of Engineering and Technology, Hyderabad-500090, India; ^5^Department of Physics, College of Science, Princess Nourah bint Abdulrahman University, P.O. Box 84428, Riyadh 11671, Saudi Arabia; ^6^Operations Research Department, Faculty of Graduate Studies for Statistical Research, Cairo University, Giza 12613, Egypt; ^7^Department of Mathematics and Actuarial Science, School of Science and Engineering, The American University in Cairo, New Cairo, Egypt

## Abstract

An electrical device that transforms the electricity into the waves of radio and vice versa is termed the antenna. Its main deployment is in the transmitter and receiver of the antenna. While transmission, the transmitter of radio at the extremities of the antenna furnishes the electricity which oscillates at the frequency of radio wave and energy is released as current as em waves. Some of the voltage is formed from the em wave that is invaded at the point of receiving to amplify the receiver. This study focuses on the analysis of the satellite system to aid in mobile antenna tracking. It also examines the techniques for fuzzy control which make up traditional networks that are used. Initially, a basic idea of tracking loops with stabilized antennas was suggested in light of the requirement for the margin of phase and bandwidth. If the gain of the track is reduced due to changes in attributes and throughput, it will be reduced. In addition, fuzzy regulators and PID constituents are used to enhance the loop. The results indicate that the higher and lower antenna tracking gains within the loop were the best fit and the loop's fluctuations are reduced. A controller based on fuzzy logic can be most efficient due to its simplicity and robustness. It is also discovered that fuzzy logic controllers are evaluated by their behavior in relation. This paper presents an evaluation of the controllers in fuzzy logic, which is based on its integration with conventional controllers. There are three gains in PID's regulator PID and every gain can be used to control the variables of inputs and outcomes. The effects of the responses were analyzed and were compared. The commonality was discovered in the results according to the increase in time for II/6 and II/3 based on PID's regulator PID stability, it can be improved by this system, and there is a reduction in the duration of stability. Furthermore, the period of stability may be reduced through the fusion of PID and fuzzy. The effectiveness of the system could be enhanced by the implementation of the neural network. It is also possible to design the two types of control that could be used to control the proposed solid platform.

## 1. Introduction

Solely antennas are implemented in every single device that makes use of radio. Their main implementation is in the broadcasting of radio, television, receivers for communication, radio of 2-way channel, mobile phones, radar, tags of RFID, wireless microphones, etc. On a standard view, an antenna is comprised of a type of positioning of metallic conductors, electrically connected to a transmitter or a receiver. A current is generating oscillations of electrons that are relayed by a transmitter via an antenna with the elements. Such fields, which fluctuate in time, are diffused into space by an antenna in the form of a transverse wave of a transverse field [1], as a reversing phenomenon of the magnetic and electric fields that oscillate the radio wave as input to the input pressure on the electrons located in the antenna which lead them to move in a to and fro motion, thus creating oscillations.

The antennas are formulated in such a manner that they receive and relay the waves of radio in all directions horizontally and equally or preferentially in a particular direction. Some extra constituents may be added to this portion providing no electricity to the receiver and transmitters like parasitic elements, parabolic reflectors, or horns, which do functions to converge which waves of radio to the beam or other desired radiation patterns. The words antenna and aerial both are the same. However, as a reference, the aerial means an antenna that is wired. The word antenna originated from the commencement of a wireless arrangement that is dedicated to Italian radio pioneer Guglielmo Marconi. In 1895, he started making trails on these long-wired so-called aerials. He found out that by picking out the aerial to a height above the ground and linking it is one extremity to the ground. Its range is enhanced. As for his fame, all scientists and the public adopted the word antenna as suggested by Marconi. While adding up to the basic working of its constituents, the antenna also gives support and closure. An antenna might be comprised of a mixer while operating at microwave frequencies [2]. To affix the connections of electricity to the field of electromagnetic, the antennas are played in the receiver of a radio. Radio waves are a type of electromagnetic wave that almost takes no loss while traveling at the speed of light in the medium of air. The receivers and transmitters of radios are played to relay the signals in systems which comprise radio for broadcasting, cellular phone, telephone, television, devices, and many more. The radio waves are played for calculation purposes in techniques that include radar, GPS, and radio astronomy. In some scenarios, the receivers and transmitters are not kept visible in antennas, but they are always there in them.

### 1.1. Reciprocity

It is the standard characteristic of an antenna so that the electrical attributes of an antenna are explained further, As an illustration, the pattern of radiation and receiving is both the same when employed for reception and when it is driven and works out as a radiator adjacently. An outcome of this is the theorem of reciprocation of electromagnetic. Thus, no difference is marked in the transmitting and receiving technology, and a view of our connivance can be obtained. The condition necessary for positive confirmation of the feature of making reciprocal elements invaded in the relay of media and the antennas is reciprocal and linear. The terminology of reciprocal is explained as the response of matter is alike in both directions if exposed to an electric or magnetic field. Most of the materials accumulate these characteristics but there is a certain material microwave in nature that possesses elements as high tech like isolators and circulators which are formulated of elements like ferrite which are of nature or nonreciprocal. Thus, the functioning of the antenna transmission and receiving is different which can be implemented in devices like radar.

## 2. Related Works

Mendel and Wu [3] presented that the main constituent of the theory of fuzzy sets is MFs and the shape plays a vital role in a defined statement of the impact of the inference system. Their shape might vary as a trapezoidal, triangle, Gaussian, etc. It is satisfied by one condition that its value must be in variance among 0-1. However, the response of MFs of Gaussian was not much good in all scenarios, and it reveals that the MFs of Gaussian are better to be deployed in the system of statics and assumptions and probabilities. Wang [4] developed the concept of a conditional fuzzy set and demonstrated that a type-2 fuzzy set and a conditional fuzzy set are identical. Moreover, we showed that the main and secondary variables, as well as the type-2 fuzzy sets, are usually independent of each other in the primary and secondary frame of the product space of the primary or secondary variables, while the second variable depends by definition on the primary variable of a type-2 fuzzy set. The conventional terminologies are much more complicated and take time. However, the controller with fuzzy logic is a better choice as it complies with the flaws of the model which is not easy to approximate as modeled. The antenna of a vehicle is a lead in a fluctuating environment but may fluctuate the attributes of the system. The traditional stratagem that is affixed to the attributes cannot lead to the satisfaction of the needs. To cover up the flaws of the traditional controller of PID, the document presented a PID controller based on fuzzy logic, which may regulate the attributes of the control of the positioning of a system of vehicle's antenna. The impact and robustness were evaluated by the simulation of the computer. The outcome obtained is to hunt the performance of the antenna of the vehicle, and the practical implementations reveal that the system with a compound control has a greater response, minimal overshoot, greater interference in antidirection, stability, and efficacy. And this conclusion can be taken out from the PID control fuzzy system that has a raise in the static performance of the system, and a required effect is attained. Ruiz-García et al. [5] presented a comprehensive framework that is available for an FLS Type-2 using the most current perception of FSs IT2, which can have secondary grades of nonconvex T1 FSs, known as the general interval type-2 fuzzy set gfIT2 FSs. That reveals the outcomes obtained from the design, study, and manufacturing of a system that solves the purpose of autotracing by satellite and searching that is deployed in the receivers of the mobile. This system is applicable for each of the conventional and fuzzy systems of PID. The first PID was formulated by Ziegler Nichols' methodology for tuning that is implemented to attain the parameters for regulation. Later, a regulator of fuzzy was implemented to regulate the controller of PID. For the implementation of the receiver of a mobile satellite, a regulatory system for the tracking purpose has been formulated that is deployed on a board of PCBs. Nguyen [6] presented the notion of bisimilarity in fuzzy DLs under the Zadeh semantics, and it is defined using the proposed notion of p-cut simulation between fuzzy interpretations. The logics under consideration are fuzzy DLs that add characteristics such as inverse roles, universal role, qualified number limitations, nominals, and the role local reflexivity to the fuzzy version of the DL ALC reg (a form of propositional dynamic logic). Zhang et al. [7] proposed a new FLS modeling framework, also called data-driven elastic FLS. Based on the DD-EFLS framework, an elastic Takagi-Sugeno-Kang (TSK) FLS modeling approach (ETSK-FLS) is suggested to train the elastic TSK FLS for modeling tasks based on high-dimensional datasets using simple rules and a more human-like inference mechanism. The suggested framework and the ETSK-FLS method's properties and benefits are empirically validated using both synthetic and real-world datasets. Eyoh et al. [8] presented a new interval type-2 Atanassov-intuitionistic fuzzy logic system (IT2AIFLS) using the Takagi-Sugeno-Kang fuzzy inference with neural network learning. It describes the modes of control of the structure of locating a system on a mobile satellite based on fuzzy and conventional methodologies of controlling that are accumulated in this examination. The margin of phase and bandwidth were the two main factors counted to design the loops for stabilization and tracking of antenna. However, if the gain in the loop of the track is minimalized because of fluctuations in attributes, the throughput will also be debased. Cintula and Diaconescu [9] proposed the omit type theorem, a key consequence of traditional representation theory, to a broad class of fuzzy logics that includes the well-known logics of left-continuous t-norms and uniform. The isolated types over a complete theory are realized in all of its models in classical logic. This is not the case in our general case; once again, it would be interesting to investigate which fuzzy logic (and for which the notion of complete theory) the claim holds. Abreu et al. [10] proposed a procedure for obtaining the residential load profile using fuzzy logic theory. Because household electrical energy consumption is strongly linked to active occupation, the number of occupants and different times of day for typical users are taken into account. Vidal et al. [11] presented a study and axiomatization of modal extensions of product fuzzy logic with semantics given by Kripke frames with crisp accessibility relations and evaluation over the canonical standard product algebra has been our main focus. Zhang [12] presented a cellular transformation of bipolar fuzzy sets to quantum intelligence(QI) machinery using an axiomatic formulation of equilibrium-based business intelligence and information conservational quantum-fuzzy cryptography (ICQFC). With mathematical, philosophical, and scientific distinctions, it is claimed that QI, as the most generic genre of intelligence, underpins artificial and biological intelligence. It is envisaged that the discipline of fuzzy sets and systems research, including but not limited to brain science and quantum information science, would rise to the forefront of modern science as a result of Beke and Kumbasar [13] who offered a systematic and interpretable design strategy for developing type-2 (T2) fuzzy logic-based linguistic pursuing strategies (PSs) and deploying them in a real-world pursuit-evasion game (PEG). The T2 fuzzy logic-based PSs have been shown to function satisfactorily versus a human user in comparative experimental findings. Vieira et al. [14] presented two antenna arrays to receive and retransmit environmental data over a nanosatellite. The main challenge was to integrate a unidirectional antenna operating in UHF onto the nanosat. The comparison of computational and measured results is shown and analyzed, with both UHF and S-band arrays achieving good performance in terms of radiation quality. Liu et al. [15] devised an adaptive fuzzy controller for both the Caputo fractional-order nonlinear system and the Riemann–Liouville fractional-order nonlinear system, both of which have uncertain system models and control gain matrices. A study conducted by Liu et al. [16] used adaptive fuzzy control to investigate the synchronization problem of fractional-order chaotic systems with input saturation and an unexplained external demand. To analyze stability, the fractional adaptation axiom is applied, and fuzzy logic systems are used to estimate anonymous nonlinear functions using fuzzy logic systems. When this control method is used to synchronize two fractional-order chaotic or hyperchaotic systems, the synchronization error approaches zero asymptotically as the number of systems increases. The authors employed two scenarios to increase the likelihood that their strategy would be successful.

The authors of [17] investigated the synchronization of two fractional-order neural networks in detail (FONN). To manage the entire actuation, one fractional-order integral sliding surface was built, and the usefulness of this method is proved by solving two linear matrix conflicts. A sliding mode controller is created using the fractional-order adaptation hypothesis to ensure that the synchronization error converging to an arbitrarily small part of the region is achieved. The article [18] investigates a resilient, optimization technique for ambiguous fractional-order financial chaotic systems with bounded uncertain external disturbances. Instability analysis changes system parameters in real-time using quadratic Lyapunov functions and fractional-order adaptation algorithms. All closed-loop signals will monotonically converge to the origin with the proposed controller.

The article [19] looks into synchronization for unspecified fractional-order neural networks with deformations. To approximate unidentified nonlinearities, fuzzy logic is used. The stability of the isolated circuit is used to construct an adaptive fuzzy synchronization controller. The authors used fractional-order adaptation laws to analyze the stability of undefined parameters using the quadratic Lyapunov function.

Synchronization for a set of unspecified fractional-order neural networks is described in [20, 21]. For online parameter updating, a dynamic synchronization controller with fractional-order Lyapunov stability is built. This functionality uses an adaptive synchronization law. The proposed controller reduces synchronization errors among two uncertain fractional-order neural networks to zero. Using novel lemmas, the stability analysis uses quadratic Lyapunov functions.

According to [22], using the fractional-order control method, synchronization for two different fractional-order chaotic systems is investigated to see if they can guarantee synchronization error while maintaining a set level of performance. The method utilizes a fractional-order synchronization controller and an adaptive fractional-order synchronization controller for fractional-order chaotic systems with and without disturbances, both of which can ensure the required version of the synchronization error for fractional-order chaotic systems. The authors of [23] use SMC and CLSMC to solve the synchronization problem of chaotic fractional-order neural networks (FONNs). They create a surface with sliding and adaptable rules. The SMC ensures that asymptotically synchronization error tends to zero under PE. To lower the rigor of the parameter prediction, online recording and immediate data are integrated. Composite learning law combines synchronization and prediction errors.

In [24], first, a supervision law is drafted. Second, the mistakes are used to estimate unknown fuzzy system parameters. Interval excitation ensures parameter convergence [25]. Classic adaptive fuzzy control design uses T–S fuzzy systems with linear rule consequences to model-controlled systems. It is possible that the system state does not match the premise variable. Incommensurate fractional-order chaotic systems with external disturbances and input saturation can be shown protectively synchronized. An adaptive controller with fractional-order parameter adaptation methods was utilized for closed-loop Mittag–Leffler stability.

## 3. Error Minimization Controlling in Antenna Position

### 3.1. Problem Statement

In order to be in charge of the state of the projection within the basic design of the project, fuzzy logic operates through. The amount that is generated is not accounted for as it requires an arrangement. The amount generated is philanthropic and has several mistakes that are not under the control of authorities. The place is calculated for Π/3 and Π/6. The location is located in the command of error, which is in the midst of 2.7 seconds for Π/6 as well as 3.2 in the case of Π/3. We need to find more clarity about the structure through the PID checker. The fuzzy logic checker's output shows the time to arrange of Π/3 as well as Π/6. It takes a long extent to get to the top of the company [26].

### 3.2. Proposed Methodology

**Figure 1:**
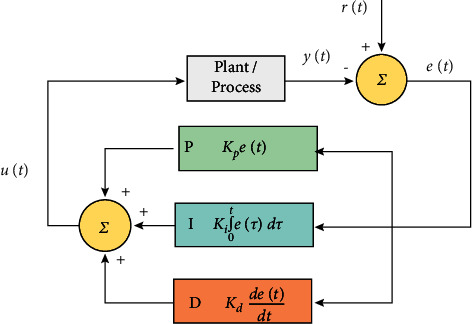
A block diagram of a controller of PID. (Source: https://en.wikipedia.org/wiki/Control_system#/media/File:PID_en_updated_feedback.svg).

A controller of the proportional–integral–derivative controller (PID) is a loop for the regulation of feedback that can be deployed over a wide area in the system of control in the industry. The value of error is computed by the controller of PID as the difference of a required point of the set and variable. The controller attempts to make the error minimal by tuning it in the process of formulation of a transforming variable.  Figure 1 shows that the controller of PID constitutes 3 attributes which are stable and are defined as integral, proportional, and derivatives abbreviated as I, P, and D. These values can be calculated as follows: P is the current (present) error, I is the errors of the past, and D is an error that will be made in future. It is constituted of variations in the current [27], and these 3 portions are implemented to formulate the regulations with the help of a regulator element as control of valve power or damper. A PSD for the scenario of discrete-time is required. The PID regulator provides more ease than others as it depends on the variables that are processed and measured but not on any information of the ongoing process. Some actions that regulate the action of needs of the process can be invaded in the regulator, by briefing these three attributes in the regulatory algorithm of PID. The points like how the regulator gives its reaction to a flaw, the extent to which a point is overhooted by the regulator, and the degree of oscillation determine how the regulator generally responds. However, the efficient control of the system is to be assured by PID. Either one or two terminologies might be sufficient to be applied in certain applications. The setting up of other attributes can attain this thing. The terms PD, P, I, or PI will be given to a PID regulator when the control actions are not there. As the derivative action possesses sensitivity toward the noise to be measured, of PI in general. However, if the main term is not there, the goal cannot be attained.

The fluctuation PID regulator has been like using the wrong control action, PID block default settings, parallel form and series tuning settings in the ISA standard form, wrong units for tuning settings, wrong units for output limits, and antireset limits tuning level controllers, violating the window of allowable controller gains. Components of a satellite consist of the communications system, which includes the antennas and transponders that receive and retransmit signals, the power system, and a “transceiver” and “transponder.” A transceiver has an independent transmitter and receiver packaged in the same unit. In a transponder, the transmit carrier frequency is derived from the received signal. The frequency linkage allows an interrogating ground station to recover the Doppler shift and thus infer range and speed from a communication signal without allocating power to a separate ranging signal.

### 3.3. PID Controller Theory

With the addition of the three terms that are part of MV, PID is given its name describing those three terms that are correcting it. To compute the outcome of a PID, the terms proportion, derivative, and integration are added up together, where *u*(*t*) is the outcome of the regulator. Thus, finally, PID can be visualized as

(1) $$\begin{array}{c} u\left(t\right)=MV\left(t\right)\\ =k_{p}e\left(t\right)+k_{i}\int_{0}^{t}e\left(\tau \right)\text{d}\tau +k_{d}\frac{\text{d}}{\text{d}t}e\left(t\right), \end{array}$$

where *k*_*p*_*k*_*p*_ is the gain in proportion, *k*_*i*_ is the gain in integration, *k*_*d*_ is the gain in derivation, *e*(*t*)=*SP* − *PV*(*t*), T is the time, and *τ* is the integration of variables. Equivalently, the function of transfer in the domain of Laplace of PID regulator is *L*(*s*)=*k*_*p*_+(*k*_*i*_/*s*) + *k*_*d*_*s*, where S is the frequency of the complex number.

**Figure 2:**
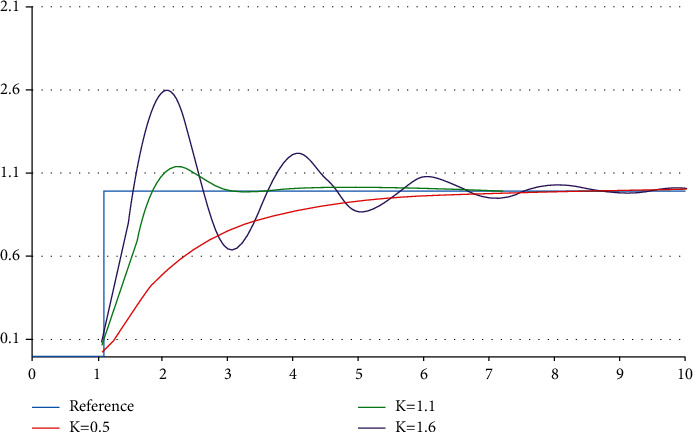
PID controller response curves.

Figure 2 shows the reaction of PV to a step-change in SP as a function of time for the three values, i.e, *k*_*p*_,  *k*_*i*_, and *k*_*d*_. The value of output generated by a term of proportion is in ratio to the present value of current. The reaction of this proportion can be tuned by the product of the error by a constant called *k*_*p*_, referred to as the proportional gain constant.

The term is described in

(2) $$\begin{array}{c} P_{\text{out}}=k_{p}e\left(t\right) \end{array}$$

For any variation in the coming flaws, an extreme variation is observed in the outcome when a gain of high proportion produces a large variation. This can lead to instability of the system if the gain of proportion is extreme. However, on the other hand, a minute gain produces a small response to the outcome with a big error in input and is not much responsive [28–30]. If the gain in proportion is not too high, in relation to fluctuations in the system, the action of control may also be small. It is clear from industrial evidence that there must be a part of the term proportion while there is a bulk change in outcome.

#### 3.3.1. Integral Term

There is proportionality in both the magnitude of a flaw that occurred and its duration in an integral term. The integral term of a PID controller outproduces the offset that needs to be rectified and is the addition of the error of an instance with time [31]. The flaw which is attained is a multiple of gain of an integral (*k*_*i*_), and then it is summed up to the outcome of the regulator.

The term of integral is sorted out as

(3) $$\begin{array}{c} I_{\text{out}}=k_{i}\int_{0}^{t}e\left(\tau \right)\text{d}\tau . \end{array}$$

**Figure 3:**
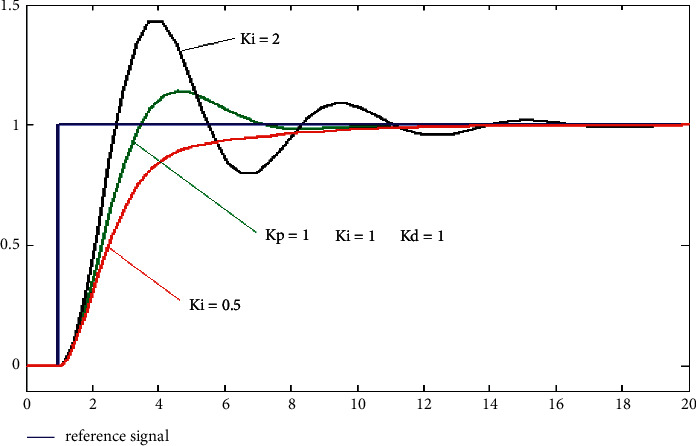
PID gains comparison.

We can see from Figure 3 that by combining the above three controllers (see Figure 1), we acquire the system's desired response curves. The error of the residue of a flow in a steady state is depleted, and the movement of the process is enhanced by the term of integration [32, 33]. Since the term of integration reacts to the flaws retained in it, the current value can be overshot to fix the value of the point.

#### 3.3.2. Derivative Term

By calculating the slope of the error to time and doing, the product of this momentum transformation with the gain of derivative *k*_*d*_ determines the derivative of error of the process. The part of the term of the derivation in the magnitude [34] of a complete process is defined as the gain of the derivation *k*_*d*_.

The term is described in (4) as follows:

(4) $$\begin{array}{c} D_{\text{out}}=K_{d}\frac{\text{d}}{\text{d}t}e\left(t\right). \end{array}$$

The working of the system is determined by the action of derivation, and thus, it helps to settle the system's stability and time. To apply the regulators of PID, there is a need to invade the filtering at a low pass, restricting the gain at high frequency and noise [35].

As the transceiver's serial port was under the control of the IC910Tester, it was not possible to use Ham Radio Deluxe for automatic Doppler shift tuning this time. The Doppler shift tuning was done manually instead. This turned out to be quite successful and no significant signal losses seemed to occur. When the satellite was approaching, the maximum frequency increase was 10 kHz, and for receding satellites, the actual frequency was 10 kHz lower than the nominal frequency value. During the pass, the frequency was 1.5 kHz.

### 3.4. Loop Tuning

For the needed reaction of control, the regulation of the attributes of its control is necessary to get the needed outcome. Various systems possess several behaviors, but stability is the most looked after. Moreover, variegated applications have various needs which might raise conflict with one another. Although there are three attributes [36] in it, the regulation of PID is a complicated task. It is a complicated task to tune a PID even there are only three attributes as it must comply with the criteria in the boundaries. Several methodologies are defined to detail the traditional modes for tuning of loops. It seems to be simple to tune a PID but is difficult to attain if there is a need for extreme stability. The performance must be upgraded but cannot be accepted with bad tuning. In general, the designs put extra care needed to be invaded until the comparison of the loop is desired. The attributes of circumstances can be defined correctly which possess nonlinearity & do not work before the project commences. The input to the process might not be stable if attributes are selected false for a PID. This may also be happened because of the gain in excess when a significant lag is present there. The stability of the margin is needed, stability of the reaction is accumulated, and there should not be any oscillation for any procedure of points of a set [37].

### 3.5. Error Minimization Controlling in Antenna Position

In the proposed system, the controller of PID and fuzzy logic is implemented for improvisation in efficacy and to minimize the rate of error. In the basic document, the controller of fuzzy logic is implemented only. As the position of the antenna is attained at the required state, the error will be computed by the controller. However, the fuzzy logic does not determine all errors at a time. And so, the controller of PID is implemented to solve this purpose. The error formulated in the stability of the signal strength which has taken about 3.2 sec for the establishment of this error rate has been corrected using PID fuzzy logic to 2.69 seconds which makes the connection stability even better than existing fuzzy logic.

### 3.6. Fuzzy PID Control Design System for Π/6

**Figure 4:**
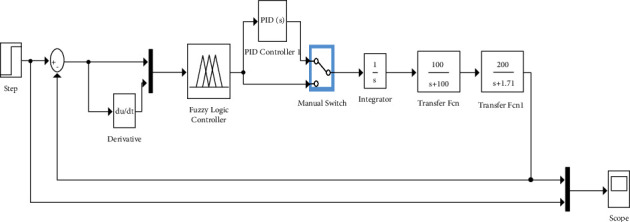
Design of the system with a controller of PID at the position of II/6.

The implementation system with a controller of PID at the position of II/6 is shown in Figure 4. The controller of PID is deployed for improvisation in efficacy and stability in time. The time was approximately 2.7 sec, but in the controller of PID, it is found to be 2.69 sec. Thus, it is observed that the working of the controller of PID is faster than that of fuzzy logic.

The gain increases when there is a raise in the time for II/6 & II/3; this affects the stability of the protocols from the satellite antenna to the transponders for the communication establishment.

### 3.7. Fuzzy PID Controller Design System for Π/3

The stability of time for the system of II/3 sec is 3.2 sec for the control of the position. The stability of the system gets into 2.7 sec for the position of II/3 controller of PID.

## 4. Results

PID (proportional integral, integral as well as derivative) numbers assist to operate the controls. The invention of the low-cost microprocessor-based PID control has eliminated most thermostat (on/off) types of devices. The majority of controllers that use microprocessors come with an autotune function that runs a system test as illustrated in Figure 2. This test helps determine the characteristics of thermal energy in the system in question. The most common technique of autotune is to input to the final control unit and then observe the output. This results in a gain directly linked to a proportional band. The delay time between the use of the input step and the perceived response affects the number of derivatives. Fuzzy Logic control is an alternative to constant adjustment of the PID parameters.

Most fuzzy logic software begins building its information base during the autotune function. The majority of the information used in the early stages of system startup comes from autotune solutions.

The integral, proportional, and derivative variables are combined to determine how much output is generated by the PID controller. There are a variety of methods for tuning the PID loop. The most effective approaches generally require the creation of a kind of process model and then selecting P I, D, and P in accordance with the parameters of the dynamic model. One of the most well-known methods is the self-tuning method. If the system has to remain active, one way to tune is to first set the Ki as well as Kd to zero and then increase Kp till the input of the loop is oscillating, and following which, it is recommended that the Kp is set to about half that amount for the “quarter decay in amplitude” kind of response. After that, increase Ki until the offset is adjusted in a sufficient amount of time to complete the process to be completed. But, too much Ki could create instability. In the end, increase Kd as needed until the loop becomes sufficient fast to return to its reference point after the disturbance of the load. But the excess Kd can cause an excessive response and an overshoot. A PID loop tuned quickly generally overshoots just a little until it reaches the setpoint quicker.

### 4.1. Fuzzy PID Controller

It is recognized that linear control systems can be controlled with precision and stability by conventional controllers. However, they have poor performance when applied for a nonlinearity [8, 98.9]. For instance, a steady-state error occurs when you apply PD controllers to systems with dead zones. To remove any steady-state error, it is suggested that the PID controller be employed to improve your steady-state errors by increasing its transient efficiency. To eliminate the issue of a strong nonlinearity system, fuzzy logic controllers are utilized that have very well-integrated transient behavior.

### 4.2. Simulation Results

Simulation results demonstrate the system's response when it is exposed to the step input having the angle 1(rad). These responses are shown for the following scenarios:

- Loop that is open and closed-loop responses
- Step response using PID controller
- Step response fuzzy controller
- Step response hybrid fuzzy-PID controller

### 4.3. Fuzzy Controller Results for Π/6

**Figure 5:**
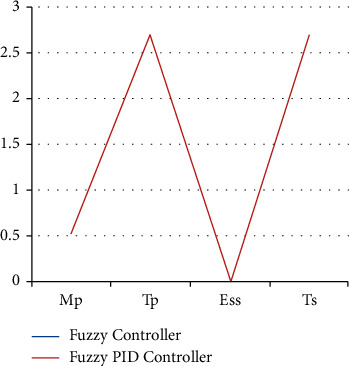
Stability graph for fuzzy logic.

In contrast to the present, the design of fuzzy logic is executed. As per the diagram, the system becomes stable after 2.7 sec.  Figure 5 presents a stability graph for fuzzy logic and time in which the line attains the needed reaction. The graph reveals that the graph attains the needed scope at 2.7 sec, which is also termed as peak time (Tp).

### 4.4. Fuzzy PID Controller Results for Π/6

**Figure 6:**
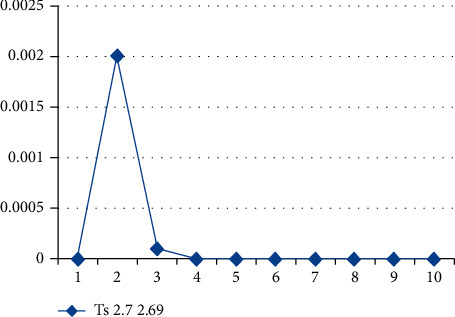
Stability graph for fuzzy PID controller.

In contrast to the present, the design of fuzzy logic is executed.  Figure 6 shows the stability of the fuzzy PID controller. As per the graph, the peak time is of 2.69 sec, and the peak time increases to 2.7 sec when the graph attains the PID. The set of times for the suggested system is minimal because of the controller design of the fuzzy logic.

### 4.5. Fuzzy Controller Results for Π/3

**Figure 7:**
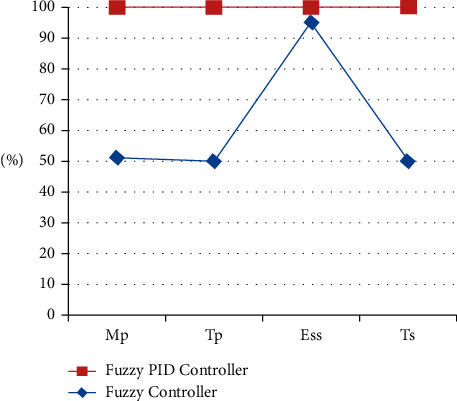
Stability graph for fuzzy logic.

In contrast to the present, the design of fuzzy logic is executed.  Figure 7 shows the stability of the fuzzy logic. As per the diagram, the system becomes stable after 3.2 sec. The graph reveals that the graph attains the needed scope at 3.3 sec, which is also termed as peak time (Tp). The related graph is shown in Figure 7.

### 4.6. Fuzzy PID Controller Results for Π/3

**Figure 8:**
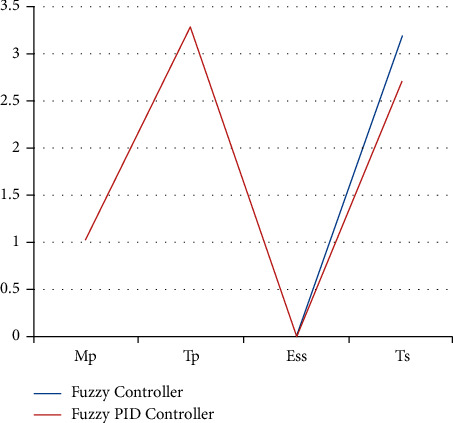
Stability graph for fuzzy PID controller.

In contrast to the present, the design of fuzzy logic is executed. As per the diagram, the system becomes stable after 3.3 sec, and the graph attains the peak time in 2.7 sec. The achieved time set for the proposed system is less than that of the fuzzy controller design.  Figure 8 shows the stability graph of the fuzzy controller and fuzzy PID controller.

**Figure 9:**
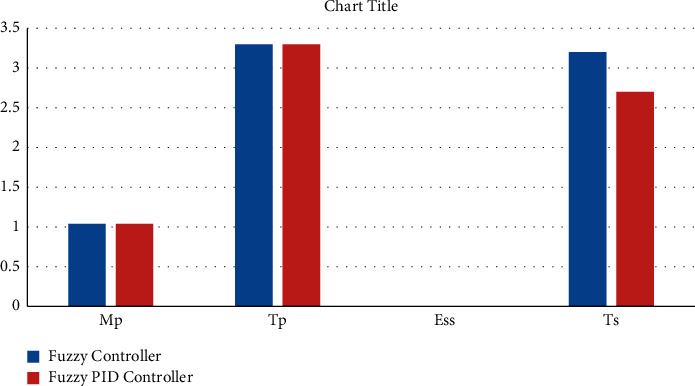
Testing parameters are drawn from the PID fuzzy model.

**Figure 10:**
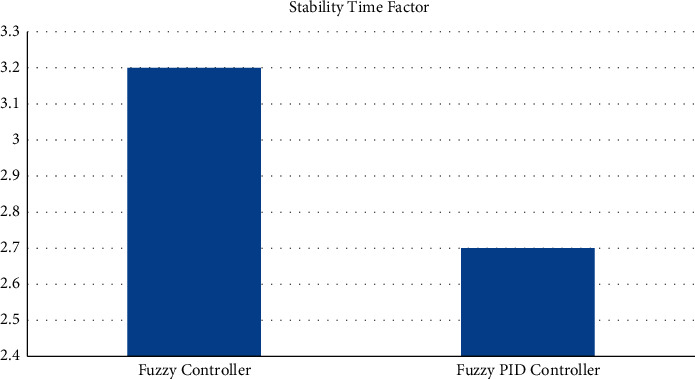
Stability time factor for fuzzy and fuzzy PID.

The test conditions are shown in Tables 1 and 2 of the stability control for existing and proposed controllers for Π/6. The parameters have been applied to the model to test the strength of the signal and its stability for two different sectors with and without PID.  Figure 9 shows the testing parameters calculated from the PID fuzzy model, and Figure 10 shows the stability time factor of fuzzy and fuzzy PID.

## 5. Conclusion and Future Scope

On a standard view, an antenna is comprised of a type of positioning of metallic conductors, electrically connected to a transmitter or a receiver. A current that is generating oscillations of electrons that are relayed by a transmitter via an antenna that fluctuates in time is diffused into space by the antenna like in the form of a transverse wave of a transverse field. The system for control of antenna of azimuth is presently available, which is discussed as the regulatory antenna of the servo by making use of potentiometers of feedback and gear. The design presently in use does not possess a controller of compensator that can furnish the controlled instability. The design of the system was assessed by S. Nise. The standard idea suggested is anyone who is commencing oversees head and shoulders and knows how to calibrate the potentiometer by hand over and shifting a larger satellite dish. The antenna of azimuth can be regulated by calibrating the angle of fire of the motor that turns the antenna, so the controller of fuzzy logic is formulated to regulate the system by adjusting the angle of fire as the outcome fluctuates. It is first stated that the controller of the PI with fuzzy logic is applied. There is one outcome with 2 inputs, and the controller observes the difference in the angle of reference and the system, and a first input termed as the error is formulated. In this research, an approach to architect controller constituted with PID is furnished to assess and put the contrasts of regulators of fuzzy logics along with the PID. There are 3 gains for the regulator of PID, and every gain can be implemented for the variables of outcomes and inputs. The outcomes of responses were evaluated and were put in contrast. It is seen that there is a similarity in the gains as per the raise in time for II/6 and II/3. As per the regulator of PID, stability is improvised by this system, and there is a reduction in the time of stability. In furtherance, the time of stability can be made minimal by the amalgamation of the controller of fuzzy and PID. The efficacy of the system can be improvised by implementing a network of neurons.

## Figures and Tables

**Table 1:** Stability control for existing and proposed controllers for Π/6.

Type	*M*_p_	*T*_p_	*E*_ss_	*T*_s_
Fuzzy controller	0.55	2.7	0.002	2.7
Fuzzy PID controller	0.52	2.7	0.0001	2.69

**Table 2:** Stability control for existing and proposed controllers for Π/6.

Type	*M*_p_	*T*_p_	*E*_ss_	*T*_s_
Fuzzy controller	1.04	3.3	0.0004	3.2
Fuzzy PID controller	1.04	3.3	0.00001	2.7

## Data Availability

The data used to support the findings of this study are included within the article.
